# The differential value of radiomics based on traditional T1-weighted sequences in newborns with hyperbilirubinemia

**DOI:** 10.1186/s12880-023-01075-6

**Published:** 2023-08-24

**Authors:** Yan Sun, Yi Liao, Fenglin Jia, Gang Ning, Xinrong Wang, Yujin Zhang, Pei Li, Haibo Qu

**Affiliations:** 1 Department of radiology, West China Second University Hospital, Sichuan University, No.1416, Section 1, Chenglong Road, 610066 Chengdu, Sichuan China; 2 Key Laboratory of Birth Defects and Related Diseases of Women and Children (Sichuan University), Ministry of Education, Chengdu, 610041 Sichuan China; 3 Bayer Healthcare Company Limited, GuangZhou, China

**Keywords:** Radiomics, Neonate, Hyperbilirubinemia, MRI, Acute bilirubin encephalopathy (ABE)

## Abstract

**Background:**

On the basis of visual-dependent reading method, radiological recognition and assessment of neonatal hyperbilirubinemia (NH) or acute bilirubin encephalopathy (ABE) on conventional magnetic resonance imaging (MRI) sequences are challenging. Prior studies had shown that radiomics was possible to characterize ABE-induced intensity and morphological changes on MRI sequences, and it has emerged as a desirable and promising future in quantitative and objective MRI data extraction. To investigate the utility of radiomics based on T1-weighted sequences for identifying neonatal ABE in patients with hyperbilirubinemia and differentiating between those with NH and the normal controls.

**Methods:**

A total of 88 patients with NH were enrolled, including 50 patients with ABE and 38 ABE-negative individuals, and 70 age-matched normal neonates were included as controls. All participants were divided into training and validation cohorts in a 7:3 ratio. Radiomics features extracted from the basal ganglia of T1-weighted sequences on magnetic resonance imaging were evaluated and selected to set up the prediction model using the K-nearest neighbour-based bagging algorithm. A receiver operating characteristic curve was plotted to assess the differentiating performance of the radiomics-based model.

**Results:**

Four of 744 radiomics features were selected for the diagnostic model of ABE. The radiomics model yielded an area under the curve (AUC) of 0.81 and 0.82 in the training and test cohorts, with accuracy, precision, sensitivity, and specificity of 0.82, 0.80, 0.91, and 0.69 and 0.78, 0.8, 0.8, and 0.75, respectively. Six radiomics features were selected in this model to distinguish those with NH from the normal controls. The AUC for the training cohort was 0.97, with an accuracy of 0.92, a precision of 0.92, a sensitivity of 0.93, and a specificity of 0.90. The performance of the radiomics model was confirmed by testing the test cohort, and the AUC, accuracy, precision, sensitivity, and specificity were 0.97, 0.92, 0.96, 0.89, and 0.95, respectively.

**Conclusions:**

The proposed radiomics model based on traditional TI-weighted sequences may be used effectively for identifying ABE and even differentiating patients with NH from the normal controls, which can provide microcosmic information beyond experience-dependent vision and potentially assist in clinical diagnosis and treatment.

## Introduction

Neonatal hyperbilirubinemia (NH) is caused by an elevated level of bilirubin, which is attributable to genetic or perinatal factors, maternal or neonatal causes, and other risk factors and markers [1–3]. Hyperbilirubinemia affects 60–80% of neonates and is the main cause of hospitalisation in the first week after birth [4–6], ranked the seventh leading cause of neonatal death within a week after birth worldwide [2], particularly in Africa and Southeast Asia [7, 8]. Physiological hyperbilirubinemia is generally harmless for most newborns, except for bilirubin levels exceeding 20 or 25 mg/dl, which are considered dangerous [8]. When the bilirubin level exceeds the bilirubin binding capacity of albumin, unconjugated bilirubin increases and potentially penetrates the blood–brain barrier, which can give rise to neurological dysfunction, termed bilirubin encephalopathy [1, 9]. If the acute manifestations of bilirubin toxicity occur within a few days of birth, acute bilirubin encephalopathy (ABE) can develop into permanent or chronic neurological damage (chronic bilirubin encephalopathy or kernicterus) [10]. In China, 8.9% of hospitalised neonates have severe hyperbilirubinemia, of which 0.9% eventually develop bilirubin encephalopathy due to a lack of proper diagnosis and prediction of its development or delayed treatment [11], which continues to lead to a disproportionately high burden.

Clinical recognition and assessment of NH or ABE are challenging. Currently, methods of measuring bilirubin and bilirubin/albumin (BA) are widely used for screening newborns for jaundice and grading hyperbilirubinemia [2]. However, it cannot directly measure the actual bilirubin level in the brain and specifically reflect the considerable nervous damage caused by bilirubin [12, 13]. Paediatricians usually judge neurological dysfunction based on patients’ early neurological symptoms and qualitative methods such as physical and neurological examination and laboratory findings, including auditory brainstem responses [14]; however, clinically, due to individual differences and treatment intervention, these changes may be non-existent or subtle, and even be overshadowed by more serious concomitant diseases.

In some cases with severe bilirubin-induced nerve damage, magnetic resonance imaging (MRI) can reflect the signal changes caused by the injury to a certain extent, which progress chronically and shifts from bilateral symmetrical hyperintensity on T1-weighted sequences to hyperintensity on T2-weighted and fluid-attenuated inversion recovery (FLAIR) sequences in specific areas of the brain [15]. However, not all patients with hyperbilirubinemia show visually observable typical imaging manifestations in existing traditional MRI sequences, which have been used clinically and frequently [16], on account of atypical imaging features of some patients and the normal myelination process in infants [17], particularly in patients not critically ill or at an early stage. Therefore, recognition of ABE or hyperbilirubinemia using MRI by traditional radiological experience-based reading methods is rather challenging in clinical practice. A variety of functional MRI techniques, including magnetic resonance spectroscopy [18] and diffusion tensor imaging [19] have been used in the quantitative analysis of bilirubin encephalopathy to attempt to address the issue of difficult qualitative diagnosis of ABE using traditional clinical MRI protocols, and some breakthroughs have been made [19, 20]. However, information on traditional MR sequences has been neglected, and few studies have been conducted using advanced image processing technology.

Radiomics, a practice for converting images into mineable quantitative data via high-throughput extraction with characterisation algorithms, has proven to be a desirable and promising future in several studies [21]. Liu et al. used a radiomics feature-based prediction model to differentiate between ABE and normal myelination in infants [22]. Therefore, taking advantage of radiomics in processing image information, we hypothesised that the combination of MRI and radiomics could deliver more quantitative information that cannot be recognised by visual-dependent reading to assist the radiological estimation of NH and the diagnosis of ABE in patients with NH.

## Materials and methods

### Patients

A total of 158 patients were enrolled in our study from February 2014 to July 2018. We enrolled 88 neonatal patients with severe hyperbilirubinemia who were admitted to the hospital for further treatment, and their cerebral MRI findings were examined during hospitalisation. The inclusion criteria for NH patients are: (1) neonates with NH; and (2) MRI images were obtained on the specific 1.5T scanner during NH period. Patients with the following conditions were excluded: (1) complications with other diseases that result in changes in MR images, such as infection, metabolic encephalopathies, congenital developmental anomalies, brain injury, etc.; (2) artefacts interfering with observation of the region of the basal ganglia; and (3)a lack of patient clinical information. Among them, 38 neonates were ABE-negative and 50 were ABE-positive diagnosed by neonatologists. ABE was defined as hazardous neonatal hyperbilirubinemia combined with symptoms such as apnea, hypotonia, reduced movement, convulsion, lethargy, irritability, hypotonous, hypertonus, feeding difficulties, or hyperthermia [3, 6].

Seventy age-matched neonates without jaundice who required cerebral MRI for clinical requirements were included in the control group. Age-matched neonates without NH must also meet the following criteria in order to be included: (1) need for clinical follow-up due to innocuous conditions like premature birth, benign prenatal abnormalities (mild lateral ventricular dilatation, small subependymal cyst, or choroid plexus cyst), or scalp hematomas caused by birth canal injury; (2) MRI images must also have been taken on the designated 1.5T scanner. Patients with the following conditions were excluded in the study: (1) complications with other diseases that result in changes in MR images, such as infection, metabolic encephalopathies, congenital developmental anomalies, brain injury, etc.; (2) basal ganglia artifacts; (3) a lack of patient clinical information.

Patient demographics and clinical characteristics, including sex, gestational age (GA), birth weight, delivery method, age at the time of MRI examination, age at admission, peak total serum bilirubin (TSB), bilirubin/albumin (B/A), length of stay, and treatments, were obtained from medical records.

### Imaging acquisition, region of interest (ROI) segmentation, and visual-based reading

#### Imaging acquisition

All images of the participants were obtained using a 1.5-T MRI scanner (Achieva Nova dual, Philips Medical Systems) using the traditional clinical brain MRI protocol, which included the sequence of axial T1-weighted spin-echo imaging (T1WI), axial and sagittal T2-weighted fast spin-echo imaging, axial T2-weighted FLAIR, and diffusion-weighted imaging. T1WI was used to construct the radiomics model in our current study with the following scanning parameters: 2000–3500 millisecond(ms) repetition time, 90–120 ms echo time, 3–5 mm(mm) thick sections, slice gap, 140–300 mm field of view, 69° flip angle and a matrix of 256 × 256.

#### ROI segmentation

T1-weighted MRI images were analysed using free and open-source ITK-SNAP software (http://www.itksnap.org/pmwiki/pmwiki.php) for semi-automatic segmentation. A region-growing method was used to sketch the whole basal ganglia as the ROI by the radiologist with five years of experience on each image slice appearing in the basal ganglia. For each subject, 4–5 image slices were included in the analysis.

**Fig. 1:**
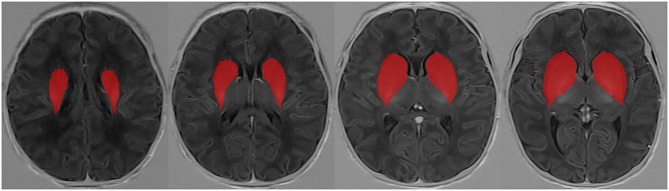
Semi-automatic segmentation. A region-growing method was used to sketch the whole basal ganglia on each image slice appearing in the basal ganglia as the ROI

Thirty subjects were randomly selected and sketched by another radiologist (10 years of pediatric radiology experience) without knowing all other information. Texture of the 30 participants’ ROI were extracted, and its consistency was analysis. The ROI drawing method is illustrated in Fig. 1.

#### Visual-based reading

All T1WI images of 88 NH patients and 70 neonates in control group were reviewed by a seasoned radiologist with 10 years of experience in paediatric imaging. He judged whether there were positive imaging changes observed on these T1 sequences of the basal ganglia using a visual-based reading strategy. All subjects were randomly assigned to radiologists who were only given access to the MRI images and no clinical data during the visual-based reading process.

### Radiomics feature extraction and feature selection

**Fig. 2:**
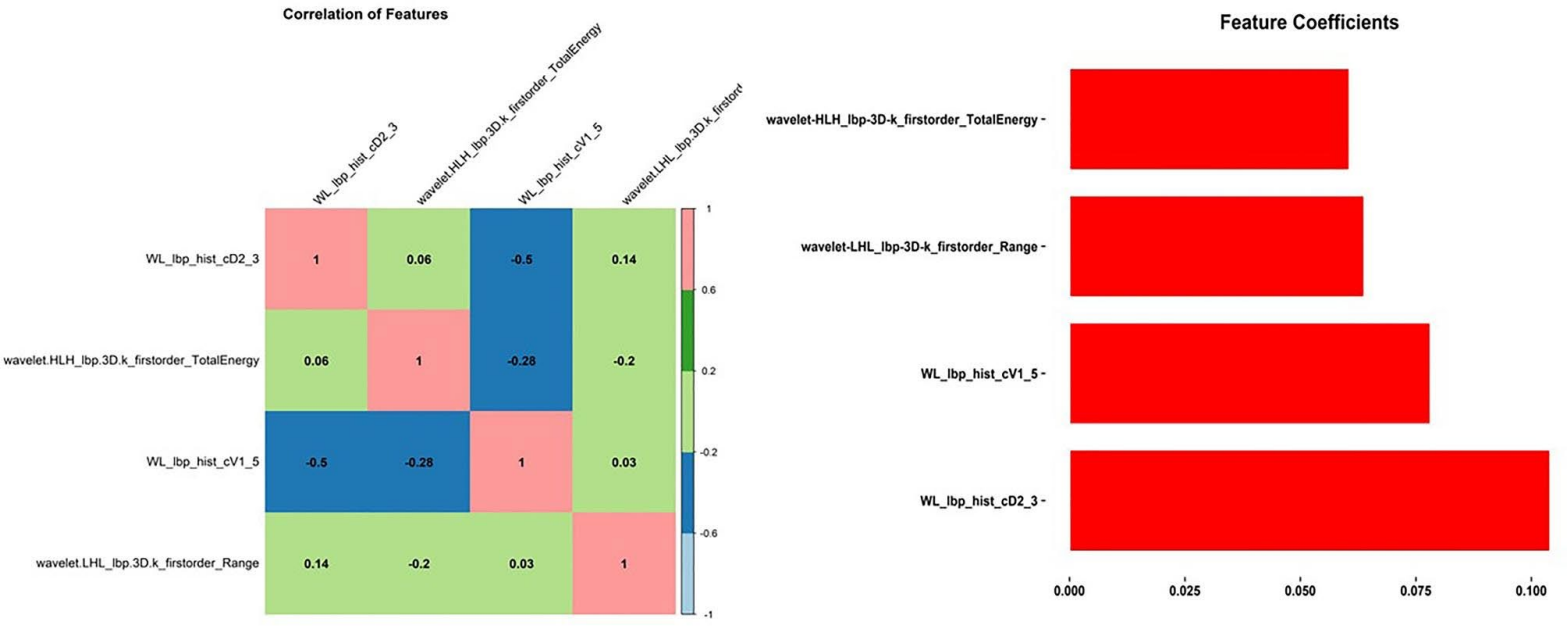
Features selection of diagnosis of ABE. The heat graph of correlation coefficient between features and bar graph of feature selection coefficient

**Fig. 3:**
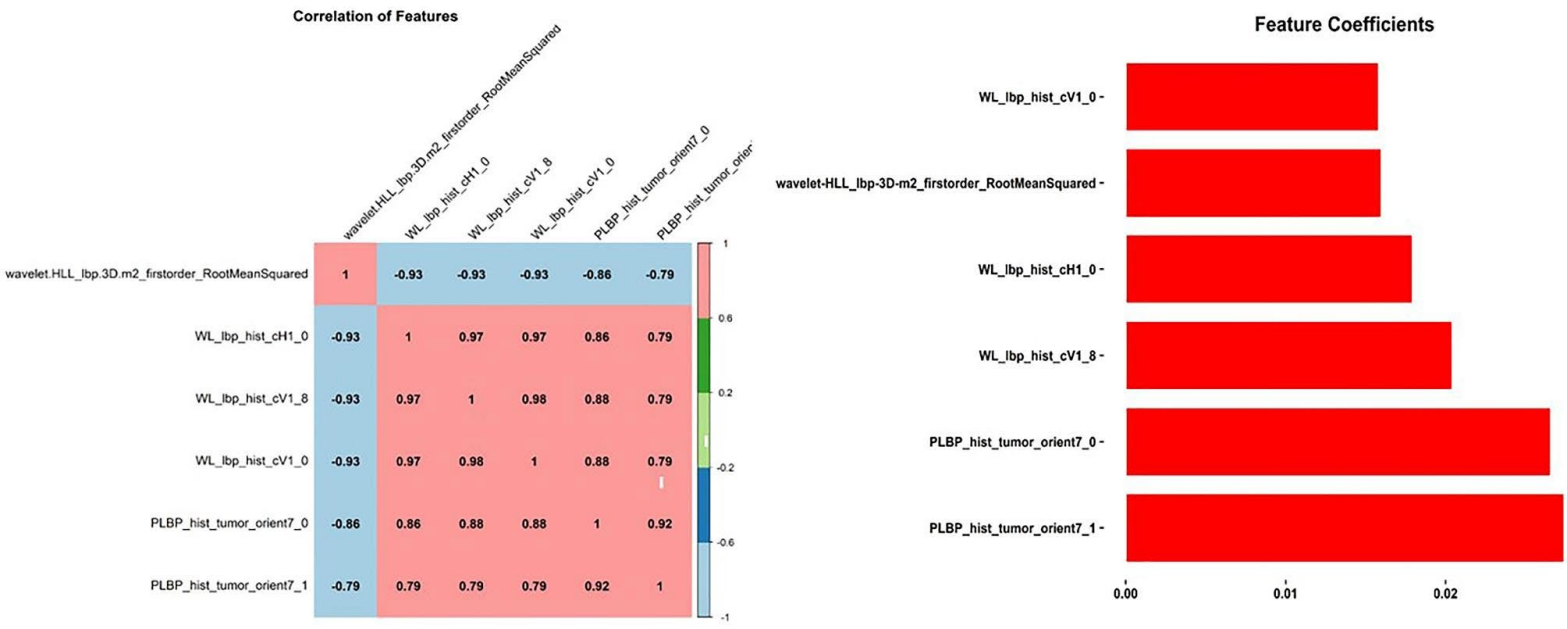
Features selection discriminating NH from the controls. The heat graph of correlation coefficient between features and bar graph of feature selection coefficient

All radiomics features were extracted using the PyRadiomics open-source package [23]. Original image data were segmented with ROI masks, and the gray level matrix of ROI were processed by intensity discretization with a default binsize of 64. No special operation was done for spatiala resampling, since the tight control of imaging acquisition, the pixel size of all images was between 0.37 and 0.57. In addition to the first-order radiomics features, we used a wavelet filter base on the original gray level matrix for wavelet radiomics features extraction, the parameters used for wavelet decomposition are shown in Table 1. A total of 744 radiomic data points were extracted from the ROI. Using stratified random sampling by class, eligible patients were divided into training and testing cohorts in a 7:3 ratio. All feature selection methods were established in the training cohort, and the results were reused in the testing cohort. Spearman’s rank correlation coefficient between the radiomics features was calculated, and for each pair of features, one was eliminated if the correlation coefficient was higher than a threshold of 0.95. Then, we used the random forest method for feature selection with a threshold of 1.25 times the importance mean. The heat maps of the correlation coefficients between the features and the feature selection coefficient bar chart are shown bellow (Figs. 2 and 3).

**Table 1:** Parameters Used for Wavelet Decomposition

Wavelet Feature Extraction	pyRadiomics
Wavelet Filtering	8 decomposition per level
Wavelet Features been Extracted	wavelet-LLH wavelet-LHL wavelet-LHH wavelet-HLL wavelet-HLH wavelet-HHL wavelet-HHH wavelet-LLL

### Statistical analysis

We used stratified sampling by class to split the original dataset into training and test cohorts, and all missing values of each feature were filled with median values. In the feature extraction step, some samples are brought into the formula, and the calculation results are too large or too small, leading to NAN values such as the denominator being extremely close to 0. We call these kinds of NAN values by missing values. Statistical analysis were performed with the software program D2G (Data to Graph software, Version 3.02; Bayer Healthcare Co.,Ltd., China). Spearman’s rank correlation was used to assess the redundancy of each pair of radiomics features. Statistical analysis was performed using IBM SPSS Statistics version 25.0. Intraclass correlation efficient (ICC) was used to consistency analysis of the ROI segmentation by two radiologists. Continuous variables are expressed as the mean ± standard deviation or median (5% *confidence interval* [*CI*], 95% *CI*) and compared using the *t*-test or Mann–Whitney *U* test. Categorical variables are expressed as numbers and percentages and compared using the χ2 test or Fisher’s exact test. Multivariate binary logistic regression was performed to build radiomics-based models, and the model was optimised using a 5-fold cross-validation method. The discrimination performance of each prediction model was quantified using the receiver operating characteristic (ROC) curve and area under the curve (AUC). Statistical significance was defined as a two-sided P-value of < 0.05.

## Results

### Participants

A total of 158 neonates were enrolled in this study, including 88 and 70 patients in the NH and control groups, respectively. Of the 88 patients with NH, 50 newborns with hyperbilirubinemia were diagnosed with ABE (ABE-positive) by neonatologists, and 38 NH patients were spared for ABE (ABE-negative). Due to the follow-up of premature newborns, 12 instances in the control group of 70 neonates required neurological MRI; however, their MRI results were normal, showing no issues with the nervous system or physical growth. Due to benign prenatal anomalies, including minor lateral ventricular dilatation, a tiny subependymal cyst, or a choroid plexus cyst, 33 neonates required postpartum MRI follow-up. Additionally, 25 cases of birth canal damage without intracerebral hemorrhage resulted in scalp hematomas.

Between the ABE-positive and ABE-negative participants, there were statistically significant differences in the delivery method (P = 0.024) and length of stay (P = 0.034). No significant differences were observed in other clinical characteristics between patients with ABE and patients with NH without ABE cohorts (Table 2), including GA, birth weight, sex, age at MRI, corrected age, age at admission, peak TSB, B/A, length of stay, and treatment methods. The GA, birth weight, sex, and age at MRI were calculated, and no significant difference was observed in the baseline data between the NH cohort and the controls, with P-values of 0.63, 0.18, 0.15, and 0.19, respectively (Table 3).

**Table 2:** Clinical characteristics of ABE-positive cohorts and ABE-negative cohorts

		Cohorts		
Information		ABE-positive	ABE-negative	*P*
Gestational Age (weeks)	Range	35.00-41.43	35.14–41.86	0.22
	Mean ± STD	38.28 ± 1.46	38.69 ± 1.54	
Birth weight (g)	Range	2200.00-5050.00	2250.00-4350.00	0.16
	Mean ± STD	3131.63 ± 499.60	3284.08 ± 509.80	
Gender	Male	25	19	1.0
	Female	25	19	
Age at MRI (weeks)	Range	0.29–3.57	0.43–3.14	0.35
	Mean ± STD	1.31 ± 0.73	1.17 ± 0.63	
Corrected Age (weeks)	Range	36.00-43.14	35.86–42.43	0.64
	Mean ± STD	39.64 ± 1.62	39.79 ± 1.37	
Mode of Delivery	Eutocia	28	30	0.024*
	Cesarean	22	8	
Age at admission (hours)	Range	26–518	26–456	0.14
	Median	160.50	125	
Peak of TSB (*µmol/L*)	Range	302.00-690.60	256.60-606.30	0.46
	Mean ± STD	455.41 ± 91.94	441.14 ± 84.43	
B/A (*mg/dl: g/l*)	Range	4.50–11.50	4.80–10.50	0.95
	Mean ± STD	7.41 ± 1.54	7.38 ± 1.31	
Length of Stay (days)	Range	4–31	3–12	0.034* (U = 701)
	Median	7	5.50	
Treatment	Phototherapy	4	4	0.72
	Phototherapy +Exchange Transfusion	46	34	

Note: Corrected Age = Gestational Age (weeks) + Age at MRI (weeks), TSB = total serum bilirubin, B/A = bilirubin/albumin

**Table 3:** Characteristics of NH patients and the healthy neonates cohorts

Characteristics		Cohorts		
		NH Patients	Control Subjects	*P*
Gestational Age (weeks)	Range	35–41	31–42	0.63
	Mean ± STD	38.50 ± 1.51	39.12 ± 2.40	
Birth weight (g)	Range	2200–5050	1410–4690	0.18
	Mean ± STD	3198.21 ± 506.90	3065.13 ± 671.10	
Gender	Male	44	43	0.15
	Female	44	27	
Age at MRI (days)	Range	2–25	1–28	0.19
	Median	7.00	8.50	

Note: NH = neonatal hyperbilirubinemia

### Consistency of the region of interest segmentation

In total, 744 original radiomic characteristics were extracted, and 677 remained after eliminating the single value, as some of which are one value in all samples, e.g., all are 0. Among them, there are 630, 7, 26, and 14 features, respectively, with ICC > 0.8, ICC=0.75 ~ 0.8, ICC~0.5 ~ 0.75, and ICC<0.5. And each potential predictor was still included in the radiomics model for feature selection, if they had a high or medium level of correlation, so we finally involved 630 features for model construction.

### Identifying ABE in patients with NH

The results of experience-dependent radiological reading showed that the expert observed the imaging characteristics of bilirubin injury in 30 (60%) of 50 ABE-positive patients. In addition, 15 (39.5%) of the 38 patients without ABE were mistakenly judged to have representative imaging of bilirubin nerve damage. The accuracy of visual-based strategy was 60.2%, and detailed diagnostic figures are shown in Table 4.

**Table 4:** Diagnostic form

Method	Clinical status		
		ABE	Without ABE
**visual-based**			
Radiodiagnosis	Positive (+)	30	15
	Negative (-)	20	23
**Radiomics-based**			
Train cohort	Positive (+)	28	11
	Negative (-)	7	15
Test cohort	Positive (+)	14	8
	Negative (-)	1	4

Note: NH = neonatal hyperbilirubinemia, ABE = acute bilirubin encephalopathy, Positive (+) = patients were diagnosed as ABE by visual-based or Radiomics-based methods, Negative (-) = patients were diagnosed as NO-ABE by visual-based or Radiomics-based methods

A total of 744 radiomics features were extracted from 88 patients with NH, including 50 newborns with ABE and 38 newborns with NH without ABE, who were divided into training and validation sets in a 7:3 ratio. After removing redundant features and features with low level consistency (ICC<0.5) feature selection was performed in 630 features and then four potential predictors were selected based on the random forest importance algorithm. These were as follows: WL_lbp_hist_cD2_4, wavelet-HLH_lbp-3D-k_firstorder_Energy, wavelet-HLH_lbp-3D-k_firstorder_TotalEnergy, and WL_lbp_hist_cD2_3.

**Fig. 4:**
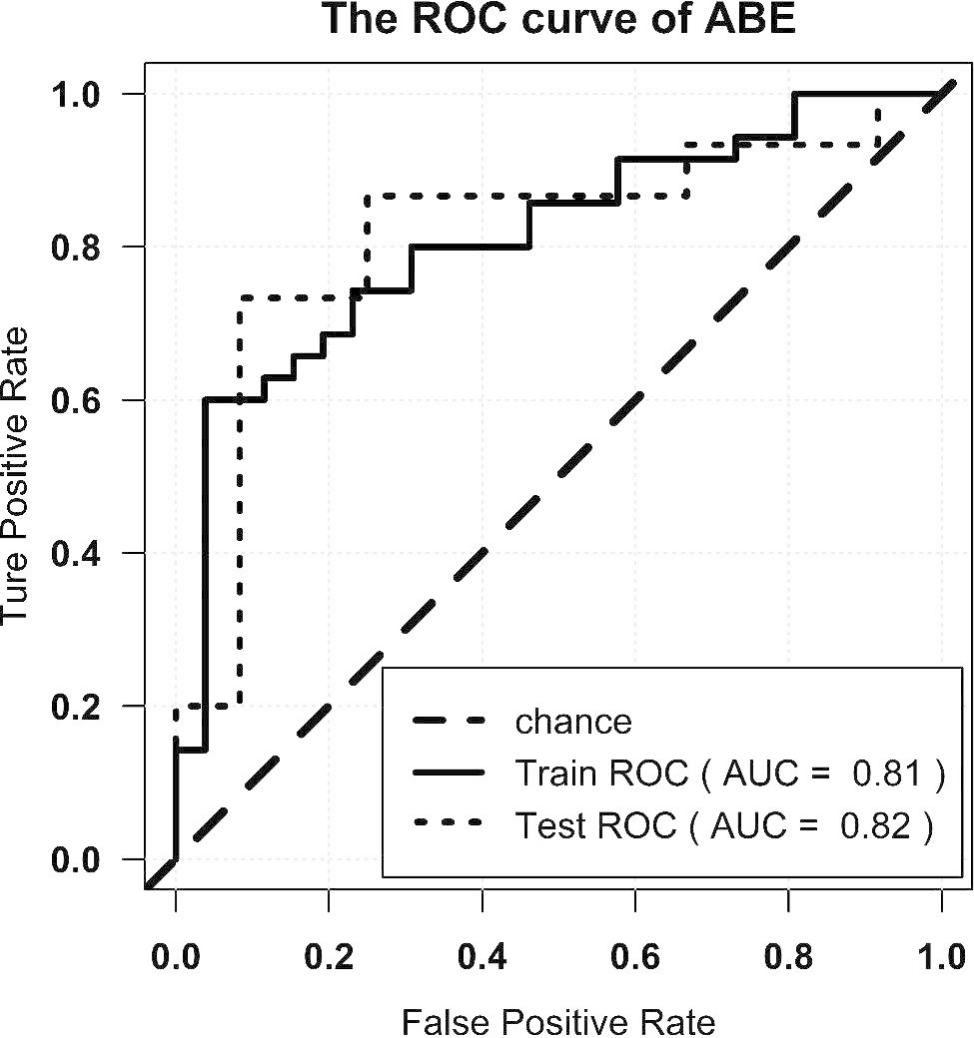
ROC curve of diagnosis of ABE. Note: ROC = receiver operator characteristic. AUC = area under the curve

The radiomics signature showed an AUC of 0.81, an accuracy of 0.82, a precision of 0.80, a sensitivity of 0.91, and a specificity of 0.69 in the primary cohort, while the validation cohort showed similar results (0.82, 0.78, 0.8, and 0.8, and 0.75, respectively). The ROC curves are shown in Fig. 4.

### Distinguishing patients with NH from the controls

All 88 NH patients (including the 30 NH newborns with ABE) had 45 (51.1%) neonates that were identified as positive, while 13 (18.6%) of the 70 normal controls were incorrectly labeled as positive patients who suffered from bilirubin injury. The accuracy of visual-based strategy was 64.6%.

A total of 88 patients with NH and 70 controls were divided into training and test cohorts with a 7:3 ratio. In total, 744 radiomics features were extracted from the images, and six were selected based on the same feature selection process mentioned previously. The radiomics features included PLBP_hist_tumour_orient7_0, WL_lbp_hist_cV1_0, PLBP_hist_tumour_orient7_1, WL_lbp_hist_cV1_8, wavelet-HLL_lbp-3D-m2_firstorder_RootMeanSquared, and WL_lbp_hist_cH1_0.

**Fig. 5:**
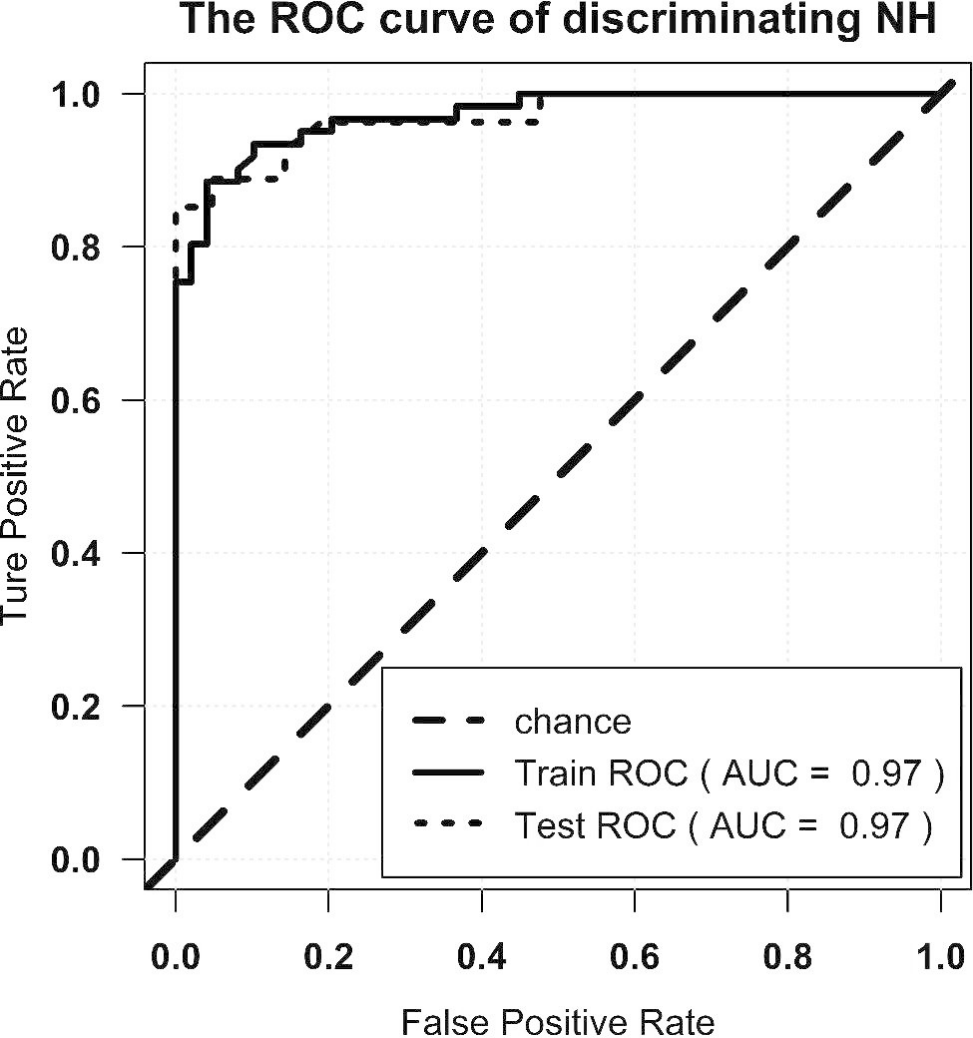
ROC curve of discriminating NH from the controls. Note: ROC = receiver operator characteristic. AUC = area under the curve

The AUC for the training cohort was 0.97, with an accuracy of 0.92, a precision of 0.92, a sensitivity of 0.93, and a specificity of 0.90. The performance of the radiomics model was confirmed by testing the test cohort. The AUC for the test cohort was 0.97, and the accuracy, precision, sensitivity, and specificity were 0.92, 0.96, 0.89, and 0.95, respectively (Fig. 5).

## Discussion

In this study, to prevent potential misidentification of basal ganglia changes in myelination such as bilirubin-induced nerve damage in neonates with a clinical history of detrimental hyperbilirubinemia exposure, the crucial data of GA and postnatal age (age at MRI) in different groups were at the same baseline to avoid the error caused by inconsistent myelination process, which was visualised as hyperintensity on T1WI sequences [17, 24, 25] due to its high lipid content. To avoid a potential impact, patients with a history of diseases which could affect the signal intensity of the basal ganglia were excluded.

In the clinical data of our study, it is worth noting that the delivery method between ABE-positive and ABE-negative patients was different, which showed that the delivery method would be a possible risk factor for ABE. This result was consistent with a previous study showing that the delivery method is a potential risk factor for severe NH [2]. Significantly, there were no statistical differences in TSB and B/A between the ABE-positive and ABE-negative groups, which was different from a previous study where TSB and B/A had potential predictive value for bilirubin encephalopathy [12], which may reflect that individual differences could affect nerve damage under the same high serum bilirubin level.Customarily, ABE-positive patients need more treatment measures and hospital observation time in clinical practice, resulting in an increased length of stay, which was in accordance with the clinical characteristics of the participants in our study. Although there are several factors leading to jaundice [2, 3], this study mainly focuses on the current MR images of neonates with hyperbilirubinemia, which were waived in our model, and the related prenatal or postnatal risk factors were not collected and analysed in detail.

Bilirubin-induced brain injury is distinctive, with remarkably selective involvement of the globus pallidus, basal ganglia, substantia nigra, hippocampus, thalamic nuclei, and putamen nuclei with a symmetric pattern [5, 16, 26]. The most common and characteristic finding of ABE was bilateral, symmetric hyperintensity on T1-weighted MRI. In our current study, as the globus pallidus without a well-defined margin could reduce the consistency of manual segmentation, we selected all vulnerable areas of the basal ganglia as the ROI on T1-weighted MRI to avoid the unclear contour to blur the accuracy of manual sketching, which was different from Liu’s research [22]. It’s important to note that the anterior internal capsule limb lies between the lentiform and caudate nuclei. The anterior internal capsule limb was incorporated into the ROI to form a whole in order to guarantee structural integrity and avoid the error produced by drawing two ROIs of the caudate nucleus and lenticular nucleus, respectively.

Hyperintensity on T1WI sequences of specific areas is the characteristic imaging manifestation of ABE; however, it cannot be denied that it is rather challenging to determine whether the high signals were due to NH [19] and even confirm the potential nerve injury in patients with hyperbilirubinemia using the current traditional radiological protocol. Although MRI has yet to become a clinical standard practice to evaluate ABE, we tried to define positive intensity using experience-dependent visual-based conventional reading strategies. In our study, an experienced paediatric radiologist could only find 34 abnormal basal ganglia in the images of 50 patients with ABE and misjudged 13 of the 38 ABE-negative patients as positive, with 60.2% findings being consistent with the clinical diagnosis. This may be due to the absence of radiological features in some ABE-positive patients, being masked by normal myelination or the slight difference being indistinguishable to the human eye. Moreover, bilirubin-induced neuronal damage or necrosis, white matter connections [5], and proliferation of microglia in the basal ganglia [27] may cause subtle changes which could be invisible, more than the abnormality of signal intensity. Therefore, we believe that traditional magnetic sequences may contain quantitative information that has not yet been discovered. Compared with advanced or functional MRI, conventional MRI protocol can shorten the examination and neonatal sedation time, increase the success of scanning, and is beneficial to the safety of infants. Accordingly, T1WI was designed to be combined with the process of radiomics in our research. In the practice of radiomics, quantitative image features reflecting the underlying pathophysiology and tumour microenvironment were extracted from digital images [28, 29].

**Fig. 6:**
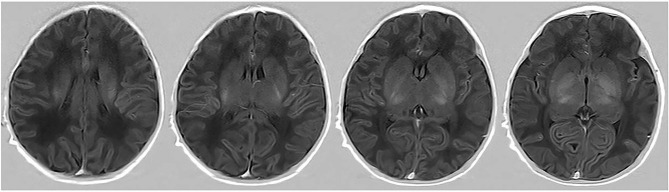
True positive case. Neonate with ABE was categorized as ABE-positive by the radiomics-based model correctly

**Fig. 7:**
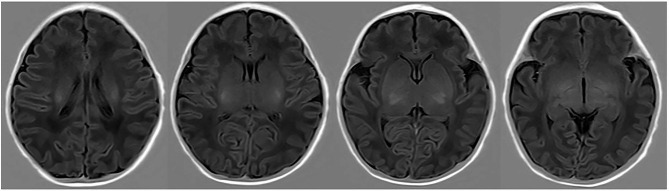
True positive case. Neonate without ABE was categorized as ABE-negative by the radiomics-based model correctly

**Fig. 8:**
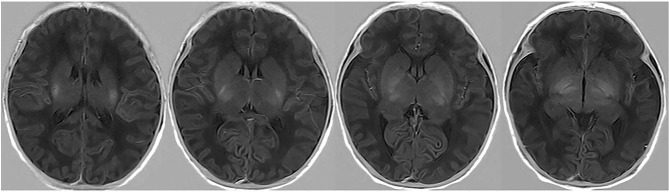
False negative case. Neonate with ABE was categorized as ABE-negative by the radiomics-based model

**Fig. 9:**
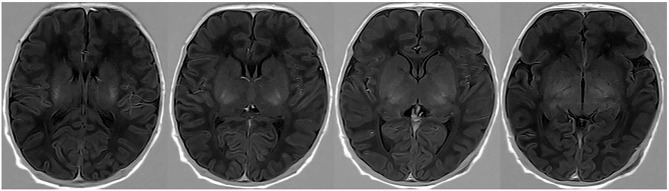
False positive case. Neonate with hyperbilirubinemia, who was spared from ABE but categorized as ABE-positive by the radiomics-based model

In 2019, Liu et al. used a radiomics approach to develop and validate a model for distinguishing ABE from normal myelination based on the T1 sequence and found that the AUC of the best classification performance was 0.946 [22]. In contrast to Liu’s research design, we included patients with hyperbilirubinemia without ABE as the control cohort instead of the normal controls in the research on the diagnosis of ABE primarily because in clinical practice, identifying patients with bilirubin-induced brain injury from patients with hyperbilirubinemia is exceedingly challenging and requires urgent attention. Four radiomics features selected from 744 were incorporated into the machine learning classification model of our current research, including wavelet and partial local binary patterns (LBP). LBP efficiently capture the local spatial patterns and greyscale contrast in the images and reflects the texture information of the region around the pixel, which was selected owing to its robustness to low-contrast and low-quality images [30], and wavelet image decomposition can remove redundant information that is not necessary [30] for diagnosing ABE in the raw image. The radiomics signature achieved AUC values of 0.81 and 0.82 for predicting ABE in patients with hyperbilirubinemia in the training and testing cohorts, respectively. The accuracy of 0.82 and 0.78 was comparable with the 0.67 accuracy of the radiologist. Radiomics with traditional MRI performed prospectively for diagnosing ABE prevails over visual-based traditional reading. Cases correctly identified by radiomics-based method were shown in Figs. 6 and 7. However, there were still some cases that could be misclassified by the radiomics-based model, as shown in Figs. 8 and 9, which were also incorrectly diagnosed using the traditional reading strategy. We believe that the small number of participants and unique T1 sequence may weaken its robustness. Hence, although improvements have been made in identifying ABE by the model, the sample size could be increased, and further exploration of potential advanced MR sequences for more useful subtle features and a more advanced deep learning algorithm for optimising the model could be performed.

Quantitative texture features identified by computational methods can further enhance the discrimination of ABE, which indirectly reflects pathophysiological changes in the brain tissue. Based on the progression of the disease, we hypothesize that even if the NH patients do not have neurological symptoms, there may already be microscopic changes in the brain caused by high levels of bilirubin, that are indistinguishable by visual identity. The individual variances and therapeutic intervention methods could result in significant differences in neurological symptoms in patients with hyperbilirubinemia, which lead to a huge challenge to accurately determine the true damage caused by high bilirubin to the nervous system. Therefore, it is particularly important to directly reflect the effects of hyperbilirubinemia on nervous system utilizing quantitative neuroimaging. If the pathological and physiological changes in brain tissue may be reflected in the bilirubin level, it is crucial for early clinical intervention of patients. Unfortunately, it is difficult to discern between NH neonates and normal infants using the conventional image reading methods. Therefore, 70 healthy newborns who were age-matched and free of jaundice were thus added to the study as the control group for analysis. In this study, only 45 out of 88 NH participants (ABE-positive and ABE-negative), including 30 NH patients with ABE who clearly displayed a signal intensity shift, could consistently distinguish from the normal using visual-based reading methods, and 13 out of 70 normal counterparts were incorrectly identified as having positive bilirubin injury. While the results of radiomics model showed that the radiomics-based machine learning approach could distinguish hyperbilirubinemia involvers from the normal controls, with an AUC of 0.97 in the training (sensitivity of 0.93 and specificity of 0.90) and validation (sensitivity of 0.89 and specificity of 0.95) sets.

However, this study has some limitations. Only a few cases from a single centre were included, and the study lacked external validation. Hence, our future study will investigate the robustness of the radiomics-based model applied to explore hyperbilirubinemia issues in multiple neonatal centres with adequate recruiters. Furthermore, this study was retrospective, and data were obtained from discharge summaries, where information could have been omitted. Lastly, the study lacked the combination of other advanced or functional MR sequences and the limitation of investigation of long-term prognosis, which is what we are working on in our future study.

In conclusion, our current study constructed and validated a radiomics-based machine learning model that estimated and diagnosed ABE and even quantitatively distinguished patients with hyperbilirubinemia from normal controls, which is superior to conventional radiological reading strategies and has the potential to provide neonatologists with clinical hints other than laboratory tests or clinical signs and symptoms.

## Data Availability

The datasets used and/or analyzed during the current study are available from the corresponding author on reasonable request.

## List of abbreviations

NH: Neonatal hyperbilirubinemia
ABE: Acute bilirubin encephalopathy
MRI: Magnetic resonance imaging
AUC: Area under the curve
BA: Bilirubin/albumin
FLAIR: Fluid-attenuated inversion recovery
GA: Gestational age
TSB: Peak total serum bilirubin
ROI: Region of interest
T1WI: T1-weighted spin-echo imaging
ms: Millisecond
mm: Millimetre
ICC: Intraclass correlation efficient
ROC: Receiver operating characteristic
LBP: Local binary patterns
